# Effects of isotemporal substitution of sedentary behavior with light-intensity or moderate-to-vigorous physical activity on cardiometabolic markers in male adolescents

**DOI:** 10.1371/journal.pone.0225856

**Published:** 2019-11-26

**Authors:** Bruno P. Moura, Rogério L. Rufino, Ricardo C. Faria, Paulo Roberto S. Amorim

**Affiliations:** 1 Medical Science Graduate Program, Medical Sciences Faculty, Rio de Janeiro State University, Rio de Janeiro, Rio de Janeiro, Brazil; 2 Department of Physical Education, Federal University of Viçosa, Viçosa, Minas Gerais, Brazil; University of New Brunswick, CANADA

## Abstract

Increasing prevalence of sedentary behavior (SB) combined with low levels of physical activity (PA) in children and adolescents has become a growing public health concern. Therefore, this study aimed to identify the daily behavioral pattern of adolescents and examine the isotemporal substitution effects of SB with light-intensity PA (LIPA) or moderate-to-vigorous PA (MVPA) on cardiometabolic markers. In this cross-sectional study, the daily behavioral pattern of Brazilian male adolescents was objectively measured for 7 days. Vector magnitude activity counts were used to estimate SB, LIPA, and MVPA with cut-points specifically validated for youth. The isotemporal substitution model was used to assess the effects of replacing different SB bouts (5, 10, 30, and 60 min) with LIPA or MVPA on cardiometabolic markers [body mass index, waist circumference, body fat percentage (BF%), total cholesterol, high-density lipoprotein cholesterol (HDL-C), non-HDL-C, low-density lipoprotein cholesterol, triglyceride (TG), glucose, insulin, homeostatic model assessment of insulin resistance (HOMA2-IR), insulin sensitivity (HOMA2-S), beta cell function (HOMA2-β), systolic-blood pressure (SBP), diastolic-blood pressure, and cardiometabolic risk score]. Male adolescents (n = 84; age, 16.7 ± 0.9 years) wore the GT3X+ for 6.7 ± 0.6 days, during 15.2 ± 2.3 h, and spent 72.9% of the time in SB, 17.3% in LIPA, and 9.8% in MVPA. SB replacement with LIPA was associated with increased HDL-C, TG, HOMA2-IR, and HOMA2-S and decreased SBP. In contrast, SB replacement with MVPA was associated with decreased BF%. Therefore, our findings suggest that replacing SB with LIPA showed positive results on HDL-C, HOMA2-S and SBP, while replacing SB with MVPA was associated with only one obesity indicator (BF%). Moreover, participants met the daily MVPA recommendations, but they still had a daily behavioral pattern with high SB. In this context, LIPAs can be considered an effective alternative to reduce SB and improve the health indicators of this population.

## Introduction

Increasing prevalence of sedentary behavior (SB) combined with low levels of physical activity (PA) in children and adolescents has become a growing public health concern [1, 2]. Objective measures indicate that adolescents spend most of their school time in sedentary activities [1–3]. SB is defined as any sitting or lying time with energy expenditure of 1.0 to 1.5 metabolic equivalents of task (METs) [4]. Conversely, PA is considered any bodily movement with an energy expenditure > 1.5 METs [5]. PA can be classified as light-intensity PA (LIPA, 1.5–3.0 METs) or moderate-to-vigorous PA (MVPA, ≥ 3.0 METs) [6]. An expanding body of evidence has suggested that SB is associated with several negative health outcomes [7–9] and is a significant cardiometabolic risk factor [7], independent of daily MVPA [10]. On the contrary, PA is an important health-related behavior [11] that provides several benefits to cardiometabolic health [12]. The effectiveness of MVPA on health is well documented and established in the literature [6, 8, 13]. However, over the last decade, due to the increasing number of epidemiological studies with accelerometers, the focus has shifted from examining the health effects of MVPA to examining health effects of LIPA [14, 15]. Although LIPA constitutes most of the daily physical activities and contributes substantially to the increase in daily energy expenditure [15], its positive effects on health are controversial [14–16].

PA is a healthy lifestyle habit [6, 11] that is prevalent in childhood but tends to decline with age [11]. To encourage the maintenance of high PA levels among adolescents and to consequently reduce the detrimental effects of SB, PA guidelines [6, 13, 17] have recommended that adolescents should perform at least 60 min·day^-1^ of MVPA. However, most PA guidelines do not explicitly recommend a reduction in SB for this population [6, 13], except for the guidelines established from countries such as Australia, Canada, and the United Kingdom [8, 17, 18].

Traditionally, multivariate regression models have been used to separately analyze the association between a behavior (e.g., SB) and health outcomes, while adjusting for other behaviors (e.g., LIPA or MVPA) as a covariate instead of replacing the time spent on this activity. Alternatively, in 2009, the isotemporal substitution model (ISM) [19] was introduced in studies regarding PA and health. This statistical technique allows a more practical approach to understand the effects of replacing the time spent on a potentially negative behavior (i.e., SB) with a potentially positive behavior (LIPA or MVPA). To date, few studies [20, 21] have used the ISM technique, proposed by Mekary et al. [19], to examine these effects on cardiometabolic markers in youth. Considering that adolescents spend more than one-third of their daily waking hours at school [22, 23] and that over 90% of this time is spent seated inside a classroom [1–3, 24, 25], understanding the impact of the interaction between different daily behaviors (SB, LIPA, and MVPA) on adolescent’s health is crucial for public health guidelines and for the development of new strategies to reduce SB and increase PA levels in this population.

Therefore, this study aimed to identify the daily behavioral pattern of male adolescents and to examine the theoretical effects of isotemporal substitution of SB with LIPA or MVPA on cardiometabolic markers.

## Materials and methods

### Study design and participants

This cross-sectional study was conducted in 2013 using a convenience sample composed of male adolescents enrolled at the Federal Institute of Education, Science, and Technology, Rio Pomba Campus, located in the southeast region of Minas Gerais State, Brazil. In 2013, the Institute had 424 students enrolled (59.2% male). Of these, 140 male adolescents resided in the student housing (The institute only provided student housing for male students). These male adolescents were considered for the study because of their similar habits, which included Institute-standardized times for waking up, studying, eating, sports, leisure, and sleeping. Researchers first met with the adolescents to introduce the research project and those meeting the following criteria were eligible to take part in the study: (a) age between 14 and 18 years and (b) provided written informed consent for participation in this study (informed consent for those aged <18 years was signed by parents or guardians). The exclusion criteria were as follows: (a) use of medication for cardiometabolic conditions, (b) previously diagnosed metabolic diseases, (c) reports of severe cardiovascular diseases or other comorbidities leading to functional disability, or (d) adolescents who are on a calorie-restricted diet. This study was conducted in accordance with the ethical principles of the Declaration of Helsinki and was approved by the Ethics Committee on Human Research of the Federal University of Viçosa (N°. 0100/2012). The datasets generated and/or analyzed during the current study are available in the Mendeley Data repository (http://dx.doi.org/10.17632/cpy375t3cp.1) [26].

### Demographic data

Age, ethnicity, and smoking status were obtained using an interview-based questionnaire. Age was determined as a continuous variable from birth to the intervention date. Ethnicity was classified into White Latin Americans or non-White Latin Americans, and smoking status (if they smoked any type of cigarette in the last 3 months) was categorized as smokers or non-smokers. Measurements of body weight (kg), height (m), waist circumference (cm), and triceps skinfold (mm) were obtained by a trained technician, according to Lohman et al. [27]. Body mass index (BMI) was calculated and subsequently categorized as normal or altered weight (overweight and/or obesity) [28]. Waist circumference (WC) was assessed at the midpoint between the last rib and the iliac crest, as recommended in previously studies [27]. Fat mass, presented as body fat percentage (BF%), was predicted by an adolescent-specific equation using the triceps skinfold [29], which was measured in duplicate with the Lange Skinfold Caliper (Beta Technology, Santa Cruz, CA). Systolic-blood pressure (SBP) and diastolic-blood pressure (DBP) were measured by a trained technician, according to standard guidelines [30]. Adolescents who presented a SBP and/or DBP above the 95^th^ percentile for their age, sex, and height were classified as having high-blood pressure [30].

### Biochemical data

Fasting blood samples (5 mL) were collected (between 6:00 and 7:00) from the median cubital vein by trained professionals. Serum glucose, total cholesterol, high-density lipoprotein cholesterol (HDL-C), and triglyceride (TG) levels were determined using the enzymatic colorimetric method. Insulin was measured using the electrochemiluminescence method. These analyses were performed using the biochemical analyzer ChemWell^®^-T (Awareness Technology^®^, Palm City, FL, US). Low-density lipoprotein cholesterol (LDL-C) and non-HDL-C were calculated according to the previous studies [31, 32]. Homeostatic model assessment of insulin resistance (HOMA2-IR), HOMA2 of insulin sensitivity (HOMA2-S), and HOMA2 of beta cell function (HOMA2-β) were assessed and calculated using The HOMA2 calculator (version 2.2.3, University of Oxford, UK) [33]. A composite cardiometabolic risk score was derived from the sum of standardized values (z-score) of the following variables: WC, average blood pressure [(SBP + DBP)/2], insulin, fasting plasma glucose, HDL-C, and TG. Subsequently, the total sum of z-scores was divided by 6 to determine the mean, which represents the cardiometabolic risk score [9, 34–36]. The higher the score, the worse the cardiometabolic status. Prior to standardization for *z*-score, natural log transformations were performed on WC, insulin, HDL-C, and TG data.

### Accelerometer data

The GT3X+ accelerometer (ActiGraph Corp., Pensacola, FL, USA) was used to estimate accelerometer valid days, daily awake time (hours), SB, LIPA, and MVPA. This activity monitor is a small device that measures triaxial acceleration (*x*, *y*, and *z* axes) within a range of ± 6 G at a sampling rate of 30 to 100 Hz and provides objective measurements of human activity with high reliability [37].

Participants were instructed to wear the accelerometer on the right side of the waist (aligned with the axillary line of the iliac crest) fixed by an elastic belt for 7 consecutive days during daily awake time and to remove it only when sleeping at night or during water-based activities (e.g., bathing or swimming) [38]. Written instructions on how to properly wear the device and the researcher’s contact information were provided to participants. All adolescents were advised not to change their daily routine. Accelerometer data were recorded at a 30 Hz sampling rate and analyzed using the ActiLife software (v6.13.3) (ActiGraph Corp., Pensacola, FL, USA). Data were reintegrated to 15-second epochs without the use of low-frequency extension filter [38].

Non-wear time was assessed using an automated algorithm, considering a minimum length of 60 min, small window length of 30 min, and spike tolerance of 2 min [39]. A valid day was defined as wear time ≥ 480 min·day^-1^ (8 h·day^-1^), and only data with at least three valid days (at least 2 weekdays and 1 weekend day) were included for further analysis [40]. The “Sleep period” and “Ignore first sedentary break of each day” options contained in the ActiLife 6 software were selected; therefore, such periods were marked as non-wear time and were excluded from further analysis. A bout length of at least 10 min and a drop time of 2 min were considered for the sedentary and PA analysis [39]. Accelerometer data from the three axes were summarized into a vector magnitude (VM) values [38, 41]. Subsequently, VM activity counts were used to estimate SB, LIPA, and MVPA with cut-points specifically validated for Brazilian adolescents (SB, ≤ 720 counts·min^-1^; LIPA, 721–3027 counts·min^-1^; and MVPA, ≥ 3028 counts·min^-1^) [38].

### Statistical analysis

All statistical analyses were performed using IBM SPSS Statistics 24 (IBM Corporation, Armonk, NY, USA), and p < 0.05 was considered statistically significant. Data normality was checked using the Shapiro-Wilk test, and due to skewed distributions, natural log transformations were performed on BMI, WC, HDL-C, TG, insulin, HOMA2-β, HOMA2-S, and HOMA2-IR. Descriptive statistics were used to summarize demographic, biochemical, physiological, and accelerometer data, which are presented as mean, standard deviation, median, and interquartile range.

Behavioral pattern was assessed by distributing of SB, LIPA, and MVPA averages in hourly blocks for each day and as averages across the valid days of the week. The behaviors recorded between 00:00 and 05:59 and those recorded between 22:00 and 23:59 were grouped on one-time block once the accelerometer was worn only during the daily awake time. In addition, physical inactivity prevalence of this sample (those not meeting MVPA guidelines, ≥ 60 min·day^-1^) [6] was checked.

A forced-entry multivariate linear regression model was used to verify the effect of isotemporal substitution [19] of SB with LIPA or MVPA on cardiometabolic markers (BMI, WC, BF%, total cholesterol, HDL-C, non-HDL-C, LDL-C, TG, glucose, insulin, HOMA2-β, HOMA2-S, HOMA2-IR, SBP, DBP, and cardiometabolic risk score). The ISM [19] was used on bouts of 5, 10, 30, and 60 min. Until recently, three bouts of at least 10-min length [13] were suggested for adults to meet the daily MVPA recommendations (≥ 30 min·day^-1^). However, for adolescents, the accumulation of at least 60 min·day^-1^ of MVPA is recommended, but there are existing evidence stating that activities performed with bouts less than 10 min have health benefits [6]. Therefore, the effects of replacing bouts of 5 to 60 min was analyzed. The statistical power for multivariate linear regression models (two-tailed, effect size = 0.2, α-error = 0.05) was based on a post hoc analysis performed using G*Power (version 3.1.9.4, University Düsseldorf, DE) [42]. All associations were adjusted for daily awake time (hours), accelerometer valid days, age, smoking status, and BMI (except when BMI was the dependent variable). Moreover, all assumptions required for multivariate linear regression were assessed, including linearity and multicollinearity.

## Results

A total of 109 male adolescents were initially considered for the study. Of these, 92 agreed to participate, but eight participants (8.7%) were excluded from further analysis due to the lack of GT3X+ accelerometer data for at least one weekend day; that is, they did not meet the established criteria for valid wear days. Thus, the present study consisted of data analysis for 84 participants. This sample size provided relevant post hoc statistical power of 0.98 for multivariate linear regression models. The demographic, biochemical, physiological, and accelerometer data are presented in Table 1. A total of 83% of the adolescents were White Latin Americans, and 95.2% of the participants were non-smokers. Overall, the participants were in good health, with mean values for biochemical markers within the normal range (except for TG, classified as borderline). Also, approximately 94% of the adolescents had normal weight and were normotensive.

**Table 1:** Demographic, biochemical, physiological, and accelerometer data for study participants. (n = 84).

	Mean	(SD)	Median	(IQR)
**Demographic**				
Age (years)	16.7	(0.9)	16.8	(1.3)
Weight (kg)	62.6	(9.5)	61.0	(9.5)
Height (m)	1.7	(0.1)	1.7	(0.1)
BMI (kg·m^-2^)	20.6	(2.9)	20.4	(2.8)
WC (cm)	73.5	(6.6)	72.0	(7.2)
BF%	24.3	(3.7)	24.1	(4.3)
**Biochemical and physiological**				
Total cholesterol (mmol/L)	4.1	(0.6)	4.1	(0.9)
HDL-C (mmol/L)	1.1	(0.2)	1.1	(0.3)
Non-HDL-C (mmol/L)	3.0	(0.6)	3.0	(0.8)
LDL-C (mmol/L)	2.5	(0.5)	2.5	(0.7)
TG (mmol/L)	1.0	(0.3)	1.0	(0.4)
Glucose (mmol/L)	4.3	(0.4)	4.3	(0.6)
Insulin (pmol/L)	51.1	(41.0)	35.2	(43.5)
HOMA2-β (%)	119.8	(70.4)	102.4	(64.8)
HOMA2-S (%)	178.6	(118.4)	156.7	(141.5)
HOMA2-IR	0.9	(0.7)	0.6	(0.8)
SBP (mmHg)	111.3	(11.2)	111.0	(16.8)
DBP (mmHg)	72.4	(7.8)	72.0	(10.8)
Cardiometabolic risk score	0.0	(0.4)	0.0	(0.5)
**Accelerometer**				
DAT (hours)	15.2	(2.3)	14.8	(2.8)
Accelerometer valid days	6.7	(0.6)	7.0	(0.0)
SB (min·day^-1^)	667.6	(129.8)	640.9	(152.0)
LIPA (min·day^-1^)	156.9	(38.3)	148.7	(52.6)
MVPA (min·day^-1^)	87.8	(22.7)	87.4	(26.0)
SB (% of DAT)	72.9	(5.7)	73.2	(8.8)
LIPA (% of DAT)	17.3	(3.8)	17.0	(6.0)
MVPA (% of DAT)	9.8	(2.8)	9.8	(3.7)

BMI, body mass index; WC, waist circumference; BF%, body fat percentage; HDL-C, high-density lipoprotein cholesterol; Non-HDL-C, non-High-density lipoprotein cholesterol; LDL-C, low-density lipoprotein cholesterol; TG, triglyceride; HOMA2-β, homeostatic model assessment of beta cell function; HOMA2-S, homeostatic model assessment of insulin sensitivity; HOMA2-IR, homeostatic model assessment of insulin resistance; SBP, systolic-blood pressure; DBP, diastolic-blood pressure; DAT, daily awake time; SB, sedentary behavior; LIPA, light-intensity physical activity; MVPA, moderate-to-vigorous physical activity; SD, standard deviation; IQR, interquartile range.

**Fig 1:**
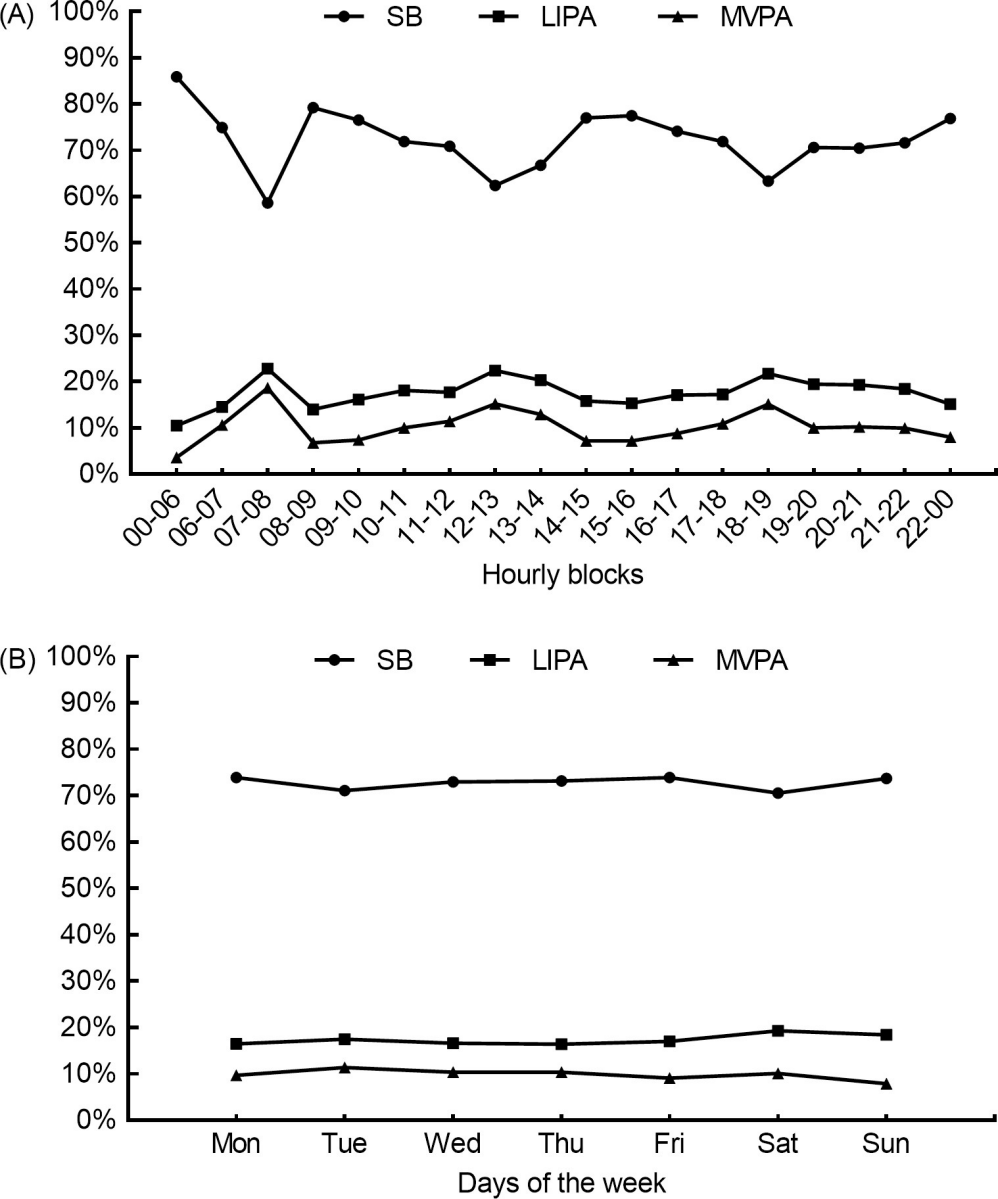
Participant daily behavioral patterns. (A) Summaries of SB, LIPA, and MVPA distributions in hourly blocks for each day. (B) Summaries of SB, LIPA, and MVPA distributions as averages across valid days of the week. SB, sedentary behavior; LIPA, light-intensity physical activity; MVPA, moderate-to-vigorous physical activity.

On average, the accelerometer was worn for 6.7 ± 0.6 days, ranging from 4 to 7 days (1.2% wore for 4 days, 5.9% for 5 days, 15.5% for 6 days, and 77.4% for 7 days), during 15.2 ± 2.3 h. Therefore, 92.9% of the accelerometer data were obtained from 6 or 7 days of wearing the activity monitor. Daily behavioral pattern consisted of high prevalence of SB (72.9%) and low prevalence of PA (17.3% of LIPA, and 9.8% of MVPA). Although these adolescents spent less than 10% of their waking hours in MVPA, 90.5% of them met daily MVPA recommendations. Fig 1A and 1B provides summaries for SB, LIPA, and MVPA distributions in hourly blocks for each day and the averages across valid days of the week.

**Fig 2:**
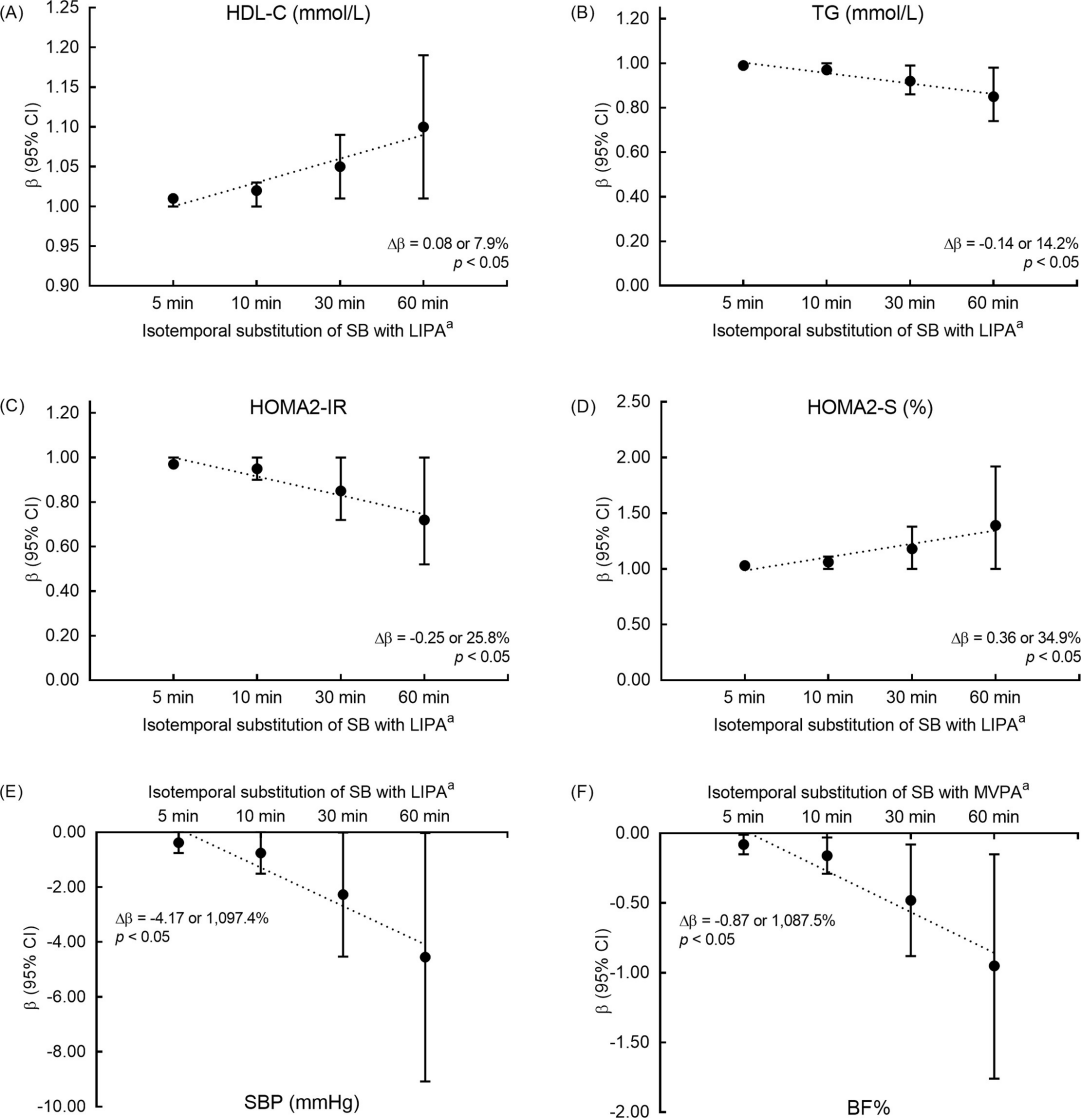
Effects of isotemporal substitution of SB with LIPA or MVPA on cardiometabolic markers. (A) HDL-C, high-density lipoprotein cholesterol. (B) TG, triglyceride. (C) HOMA2-IR, homeostatic model assessment of insulin resistance. (D) HOMA2-S, homeostatic model assessment of insulin sensitivity. (E) SBP, systolic-blood pressure. (F) BF%, body fat percentage; SB, sedentary behavior; LIPA, light-intensity physical activity; MVPA, moderate-to-vigorous physical activity; CI, confidence interval.^a^Adjusted for daily awake time (hours), accelerometer valid days, age, smoking status, and body mass index (BMI) (except when BMI was the dependent variable).HDL-C, TG, HOMA2-IR, and HOMA2-S data were transformed from natural log scale for better interpretation.Δβ = β_60_ - β_5_ and % represents the increase or decrease from β_5_.Dotted line represents the linear trend over all bouts.

The ISM results showed that LIPA and MVPA were associated with different cardiometabolic markers, as shown in S1 and S2 Tables. Fig 2 demonstrates the effects of isotemporal replacement of SB with LIPA or MVPA only for statistically significant variables. SB replacement with LIPA was associated with increased HDL-C, TG, HOMA2-IR, and HOMA2-S and decreased SBP. In contrast, SB replacement with MVPA was associated with decreased in BF%. No statistical significance was observed for BMI, WC, total cholesterol, non-HDL-C, LDL-C, glucose, insulin, HOMA2-β, DBP, and cardiometabolic risk score (S1 and S2 Tables).

## Discussion

In this study, daily behaviors were measured with a triaxial accelerometer, and its components (SB, LIPA, and MVPA) were estimated by VM activity count using cut-points specifically validated for adolescents [38]. Moreover, the ISM was used to examine the theoretical effects of replacing SB with LIPA or MVPA on cardiometabolic markers of male adolescents.

The ISM results suggest that the replacement of SB with LIPA or MVPA is associated with different cardiometabolic markers. Replacing SB with LIPA significantly promotes positive outcomes on metabolic (HDL-C and HOMA2-S) and physiological (SBP) indicators, whereas reducing SB with MVPA was associated with only one obesity indicator (BF%). Nevertheless, for the data in this sample, SB replacement with LIPA resulted in adverse effects on TG and HOMA2-IR. In the analysis of TG and HOMA-IR, it was observed that the replacement of SB with LIPA led to an increase in serum levels of those variables. This result was relatively unexpected as it was assumed that enzymatic actions triggered by muscle contractions resulting from LIPAs would promote a reduction in TG and HOMA-IR levels [14]. Therefore, it should be noted that the TG baseline was labeled as borderline, and this may have influenced this outcome. However, when observing the theoretical changes in TG and HOMA-IR resulting from the isotemporal replacement of SB with LIPA over different bout durations (from 5 min to 60 min), linear reduction in the levels of those markers was observed. But even then, the effects were still adverse, although the changes were attenuated with the greater volume of time reallocation.

To the best of our knowledge, only two studies [20, 21] used the ISM, proposed by Mekary et al. [19], to examine the association between SB and PA with cardiometabolic markers as outcome variables in adolescents. Moore et al. [20] investigated the effects of replacing time spent on LIPA with vigorous PA and reported a beneficial association on insulin levels. In another study, Hansen et al. [21] identified that replacing SB with LIPA or MVPA resulted in beneficial associations on several cardiometabolic risk factors (WC, SBP, LDL-C, insulin, TG, and glucose). Although these studies [20, 21] used ISM, only Hansen et al. [21] showed similarities with the model applied in the present study.

These aforementioned studies [20, 21] present some methodological characteristics that should be noted, such as the use of secondary data integrated from an international database [43]. These data were collected using a uniaxial ActiGraph accelerometer, with different criteria for accelerometer wear time validation and cut-point thresholds. While these studies [20, 21] had different sample sizes, it is likely that the data from the original studies were used in both cases. Therefore, it is possible that these data could have a large intrinsic variation since they come from distant countries with different diets, cultures, and habits. These factors may likely have influenced the findings from such studies. Consequently, the existence of methodological differences between the studies makes it difficult to compare the results and could explain some differences compared to our findings. Nevertheless, we verify that our results follow the same trend identified by Hansen et al. [21], that is, LIPA and MVPA have different effects on various cardiometabolic markers.

Moreover, our results were similar to that of the previous studies [9, 12, 16] conducted with adults, which indicate that the replacement of SB with LIPA significantly promote positive outcomes on HDL-C, HOMA2-S, and SBP. Conversely, replacement of SB with MVPA was only associated with one obesity indicator (BF%). Although the present study did not investigate the dose-response relationship between SB replacement with PA and health outcomes. Yet, our findings indicated significant health benefits of replacing as little as 5 min of SB with 5 min of LIPA or MVPA, consistent with the results of the previous studies [6, 44], in which activities with bouts lasting less than 10 min were able to promote positive health outcomes. This result should not be interpreted as the minimum amount of PA that should be performed throughout the day as the magnitude of the benefits achieved with 5 min bouts may not be clinically significant. However, we must consider that performing several 5 min bouts throughout the day may contribute to increasing the total amount of PA and consequently lead to an increase in the total daily energy expenditure. Additionally, it is important to highlight that the health benefits of LIPA are not greater than those of MVPA. Thus, the approach used here was a simulation and recommendations to perform LIPA should be in addition to MVPA. The potential health benefits of LIPA should be interpreted as a window of opportunity to change behaviors, because it provides a feasible alternative for reducing engagement in daily SB.

We emphasize that the magnitude of health benefits increases with greater time spent in LIPA and MVPA at different combinations. Thus, considering that adolescents spend many hours in sedentary activities at school [1–3, 22–24], searching for new alternatives to reduce harmful health outcomes caused by the high prevalence of SB combined with low daily PA rates is required. Therefore, the clinical message that should prevail seems to be simple and straightforward: every step counts and even LIPA seems to be beneficial [45, 46].

Our results also showed that 90.5% of these adolescents met MVPA daily recommendations [6], but they still had a daily behavioral pattern with high SB values. This diagnosis is concerning because the daily SB pattern presented by these adolescents is very similar to behavioral pattern shown by the UK’s older adults, who spend 73% of the day in SB and 27.5% in PA (23% in LIPA and 4.5% in MVPA) [47]. These findings indicate the need for elaboration of strategies by politicians, public managers, educators, and healthcare professionals to significantly reduce SB. A sedentary adolescent will most likely be a sedentary adult, vulnerable to serious health problems [6, 11]. This results in an increasing burden on the health and social security system. When analyzing the PA components, we found that LIPA was the most prevalent during daily awake time, with approximately twice the time spent on MVPA. Thus, LIPA may be an effective alternative to help reduce SB [14, 16]. In this context, the challenge for public health professionals, policy makers, teachers, and principals will be to develop ways of limiting sedentary time and increasing activity at any level, especially within the school environment, as this may considerably improve health and reduce mortality at the long term in the general population [45, 46].

Some studies [14, 15] have suggested that the physiological mechanisms related to LIPA benefits may be due to increased activity of lipoprotein lipase and hormone-sensitive lipase that regulates lipid metabolism following muscle contractions, leading to breakdown of TGs into free fatty acid, reducing circulating TG levels [14]. This fact may also explain the linear reduction observed for TG when the replacement time was increased from 5 to 60 min. Free fatty acids are the main fuel for slow-twitch muscle fibers (type I), which have high oxidative and low glycolytic capacities [14]. These muscle fibers are relatively resistant to fatigue and are predominantly recruited during LIPAs [15]. Moreover, there are existing evidence stating that even small increases in contractile activity may significantly increase the expression of muscle glucose transporters (glucose transporter (GLUT)-1 and GLUT-4) and glucose tolerance in sedentary individuals [48], which could explain the changes in HOMA-IR and HOMA-S.

The present study has the following strengths: (a) the objective measurement of daily behaviors with a triaxial accelerometer that provides highly reliable measurements [37] and (b) the use of the isotemporal substitution approach [19, 49] to examine the theoretical effects on cardiometabolic markers when replacing SB with LIPA or MVPA. However, this study has some limitations that should be considered. First, as in all cross-sectional studies, the causality of the effects for the observed associations cannot be determined. Second, although this study has adequate statistical power for multivariate linear regression test, its relatively small sample size may have affected the magnitude of the results and the detection of significant associations for some outcomes. Third, the TG baseline, which was classified as borderline, may have an impact on the associations of this variable. Fourth, the inclusion of only male adolescents in our sample limits the generalization of outcomes.

## Conclusions

Our findings suggest that the replacement of SB with LIPA or MVPA is associated with different cardiometabolic markers in male adolescents. Replacing SB with LIPA showed positive results on metabolic (HDL-C and HOMA2-S) and physiological (SBP) indicators, while replacing SB with MVPA was only associated with one obesity indicator (BF%). Moreover, our results also indicated that male adolescents met the daily MVPA recommendations, but they still had a daily behavioral pattern with high SB. In this context, LIPAs may be an effective alternative to reduce SB and improve the health indicators of this population. However, further studies addressing the ISM, which include 24-h data collection protocols in female and male adolescents, contemplating different experimental designs, are required to understand the issues related to behavioral co-dependency between SB, different PA intensities, and sleep.

## Supporting information

S1 Table Effects of isotemporal substitution of SB with LIPA on cardiometabolic markers. (PDF)

S2 Table Effects of isotemporal substitution of SB with MVPA on cardiometabolic markers. (PDF)
