# Supplementary material for: Effects of isotemporal substitution of sedentary behavior with light-intensity or moderate-to-vigorous physical activity on cardiometabolic markers in male adolescents
Source: PLoS One. 2019 Nov 26;14(11):e0225856. doi: 10.1371/journal.pone.0225856 (PMC6879145; doi:10.1371/journal.pone.0225856)
Supplement: S1 Table — (PDF) [file pone.0225856.s001.pdf]

**S1 Table. Effects of isotemporal substitution of SB with LIPA on cardiometabolic markers.**

| Variables | Substituting SB | with LIPA [a] | β | Lower 95% CI | Upper 95% CI |
|---|---|---|---|---|---|
| BMI (kg·m$^{-2}$) [b] | 05 min | 05 min | 1.00 | 1.00 | 1.01 |
| | 10 min | 10 min | 1.00 | 0.99 | 1.01 |
| | 30 min | 30 min | 1.00 | 0.97 | 1.03 |
| | 60 min | 60 min | 1.00 | 0.94 | 1.06 |
| WC (cm) [b] | 05 min | 05 min | 1.00 | 1.00 | 1.00 |
| | 10 min | 10 min | 1.00 | 1.00 | 1.01 |
| | 30 min | 30 min | 1.00 | 0.99 | 1.02 |
| | 60 min | 60 min | 1.01 | 0.98 | 1.04 |
| BF% | 05 min | 05 min | 0.01 | -0.03 | 0.05 |
| | 10 min | 10 min | 0.02 | -0.07 | 0.11 |
| | 30 min | 30 min | 0.06 | -0.20 | 0.32 |
| | 60 min | 60 min | 0.12 | -0.39 | -0.64 |
| Total cholesterol (mmol/L) | 05 min | 05 min | -0.40 | -1.29 | 0.49 |
| | 10 min | 10 min | -0.80 | -2.58 | 0.99 |
| | 30 min | 30 min | -2.39 | -7.73 | 2.96 |
| | 60 min | 60 min | -4.63 | -15.34 | 6.08 |
| HDL-C (mmol/L) [b] | 05 min | 05 min | **1.01** | **1.00** | **1.01** |
| | 10 min | 10 min | **1.02** | **1.00** | **1.03** |
| | 30 min | 30 min | **1.05** | **1.01** | **1.09** |
| | 60 min | 60 min | **1.10** | **1.01** | **1.19** |
| Non-HDL-C (mmol/L) | 05 min | 05 min | -0.70 | -1.54 | 0.15 |
| | 10 min | 10 min | -1.40 | -3.09 | 0.30 |
| | 30 min | 30 min | -4.19 | -9.26 | 0.89 |
| | 60 min | 60 min | -8.27 | -18.43 | 1.89 |
| LDL-C (mmol/L) | 05 min | 05 min | -0.43 | -1.16 | 0.31 |
| | 10 min | 10 min | -0.85 | -2.33 | 0.63 |
| | 30 min | 30 min | -2.55 | -6.98 | 1.89 |
| | 60 min | 60 min | -5.02 | -13.89 | 3.85 |
| TG (mmol/L) [b] | 05 min | 05 min | **0.99** | **0.98** | **1.00** |
| | 10 min | 10 min | **0.97** | **0.95** | **1.00** |
| | 30 min | 30 min | **0.92** | **0.86** | **0.99** |
| | 60 min | 60 min | **0.85** | **0.74** | **0.98** |
| Glucose (mmol/L) | 05 min | 05 min | -0.12 | -0.39 | 0.16 |
| | 10 min | 10 min | -0.23 | -0.79 | 0.32 |
| | 30 min | 30 min | -0.70 | -2.37 | 0.97 |
| | 60 min | 60 min | -1.34 | -4.69 | 2.02 |
| Insulin (pmol/L) [b] | 05 min | 05 min | 0.97 | 0.95 | 1.00 |
| | 10 min | 10 min | 0.95 | 0.90 | 1.00 |
| | 30 min | 30 min | 0.85 | 0.72 | 1.00 |
| | 60 min | 60 min | 0.72 | 0.52 | 1.00 |
| HOMA2-IR [b] | 05 min | 05 min | **0.97** | **0.95** | **1.00** |
| | 10 min | 10 min | **0.95** | **0.90** | **1.00** |
| | 30 min | 30 min | **0.85** | **0.72** | **1.00** |
| | 60 min | 60 min | **0.72** | **0.52** | **1.00** |

| | | | | | |
|---|---|---|---|---|---|
| HOMA2-S (%) [b] | 05 min | 05 min | **1.03** | **1.00** | **1.06** |
| | 10 min | 10 min | **1.06** | **1.00** | **1.11** |
| | 30 min | 30 min | **1.18** | **1.00** | **1.38** |
| | 60 min | 60 min | **1.39** | **1.00** | **1.92** |
| HOMA2-β (%) [b] | 05 min | 05 min | 0.99 | 0.97 | 1.00 |
| | 10 min | 10 min | 0.97 | 0.93 | 1.01 |
| | 30 min | 30 min | 0.91 | 0.81 | 1.02 |
| | 60 min | 60 min | 0.83 | 0.66 | 1.04 |
| SBP (mmHg) | 05 min | 05 min | **-0.38** | **-0.76** | **0.00** |
| | 10 min | 10 min | **-0.76** | **-1.51** | **0.00** |
| | 30 min | 30 min | **-2.27** | **-4.53** | **-0.01** |
| | 60 min | 60 min | **-4.55** | **-9.08** | **-0.03** |
| DBP (mmHg) | 05 min | 05 min | -0.13 | -0.41 | 0.15 |
| | 10 min | 10 min | -0.26 | -0.82 | 0.31 |
| | 30 min | 30 min | -0.77 | -2.47 | 0.92 |
| | 60 min | 60 min | -1.57 | -4.96 | 1.82 |
| CRS | 05 min | 05 min | -0.01 | -0.03 | 0.00 |
| | 10 min | 10 min | -0.02 | -0.05 | 0.01 |
| | 30 min | 30 min | -0.07 | -0.16 | 0.02 |
| | 60 min | 60 min | -0.14 | -0.32 | 0.03 |

SB, sedentary behavior; LIPA, light-intensity physical activity; BMI, body mass index; WC, waist circumference; BF%, body fat percentage; HDL-C, high-density lipoprotein cholesterol; Non-HDL-C, non-high-density lipoprotein cholesterol; LDL-C, low-density lipoprotein cholesterol; TG, triglyceride; HOMA2-IR, homeostatic model assessment of insulin resistance; HOMA2-S, homeostatic model assessment of insulin sensitivity; HOMA2-β, homeostatic model assessment of beta cell function; SBP, systolic-blood pressure; DBP, diastolic-blood pressure; CRS, cardiometabolic risk score; CI, confidence interval. Boldface represents the statistical significance ($p < 0.05$).

[a]Adjusted for daily awake time (hours), accelerometer valid days, age, smoking status, and body mass index (BMI) (except when BMI was the dependent variable).

[b]Data transformed from natural log scale for better interpretation.
