# Supplementary material for: Effects of isotemporal substitution of sedentary behavior with light-intensity or moderate-to-vigorous physical activity on cardiometabolic markers in male adolescents
Source: PLoS One. 2019 Nov 26;14(11):e0225856. doi: 10.1371/journal.pone.0225856 (PMC6879145; doi:10.1371/journal.pone.0225856)
Supplement: S2 Table — (PDF) [file pone.0225856.s002.pdf]

S2 Table. Effects of isotemporal substitution of SB with MVPA on cardiometabolic markers.

Variables	Substituting SB	with MVPA [a]	β	Lower 95% CI	Upper 95% CI
BMI ($kg \cdot m^{-2}$) [b]	05 min	05 min	1.00	0.99	1.01
	10 min	10 min	1.00	0.99	1.02
	30 min	30 min	1.01	0.96	1.06
	60 min	60 min	1.02	0.93	1.12
WC (cm) [b]	05 min	05 min	1.00	1.00	1.00
	10 min	10 min	1.00	0.99	1.01
	30 min	30 min	0.99	0.97	1.01
	60 min	60 min	0.99	0.95	1.03
BF%	05 min	05 min	**-0.08**	**-0.15**	**-0.01**
	10 min	10 min	**-0.16**	**-0.29**	**-0.03**
	30 min	30 min	**-0.48**	**-0.88**	**-0.08**
	60 min	60 min	**-0.95**	**-1.76**	**-0.15**
Total cholesterol (mmol/L)	05 min	05 min	1.11	-0.28	2.50
	10 min	10 min	2.21	-0.57	4.99
	30 min	30 min	6.64	-1.70	14.98
	60 min	60 min	12.92	-3.82	29.66
HDL-C (mmol/L) [b]	05 min	05 min	1.00	0.99	1.01
	10 min	10 min	0.99	0.97	1.02
	30 min	30 min	0.98	0.92	1.05
	60 min	60 min	0.96	0.85	1.09
Non-HDL-C (mmol/L)	05 min	05 min	1.19	-0.13	2.51
	10 min	10 min	2.39	-0.25	5.03
	30 min	30 min	7.16	-0.76	15.08
	60 min	60 min	14.05	-1.83	29.93
LDL-C (mmol/L)	05 min	05 min	1.07	-0.08	2.23
	10 min	10 min	2.15	-0.16	4.45
	30 min	30 min	6.44	-0.48	13.36
	60 min	60 min	12.71	-1.15	26.58
TG (mmol/L) [b]	05 min	05 min	1.01	0.99	1.03
	10 min	10 min	1.01	0.98	1.05
	30 min	30 min	1.04	0.93	1.16
	60 min	60 min	1.08	0.87	1.34
Glucose (mmol/L)	05 min	05 min	0.11	-0.33	0.54
	10 min	10 min	0.21	-0.66	1.08
	30 min	30 min	0.63	-1.97	3.23
	60 min	60 min	1.09	-4.15	6.33
Insulin (pmol/L) [b]	05 min	05 min	1.03	0.99	1.07
	10 min	10 min	1.06	0.97	1.16
	30 min	30 min	1.20	0.93	1.54
	60 min	60 min	1.45	0.87	2.42
HOMA2-IR [b]	05 min	05 min	1.03	0.99	1.07
	10 min	10 min	1.06	0.98	1.15
	30 min	30 min	1.19	0.93	1.54
	60 min	60 min	1.45	0.87	2.40

HOMA2-S (%) [b]	05 min	05 min	0.97	0.93	1.01
	10 min	10 min	0.94	0.87	1.03
	30 min	30 min	0.84	0.65	1.08
	60 min	60 min	0.69	0.42	1.15
HOMA2-β (%) [b]	05 min	05 min	1.02	0.99	1.05
	10 min	10 min	1.03	0.98	1.10
	30 min	30 min	1.11	0.93	1.32
	60 min	60 min	1.24	0.87	1.77
SBP (mmHg)	05 min	05 min	0.33	-0.26	0.91
	10 min	10 min	0.65	-0.53	1.83
	30 min	30 min	1.95	-1.58	5.49
	60 min	60 min	4.00	-3.07	11.08
DBP (mmHg)	05 min	05 min	0.12	-0.32	0.57
	10 min	10 min	0.25	-0.64	1.13
	30 min	30 min	0.74	-1.91	3.39
	60 min	60 min	1.43	-3.87	6.73
CRS	05 min	05 min	0.01	-0.01	0.03
	10 min	10 min	0.02	-0.02	0.07
	30 min	30 min	0.07	-0.07	0.21
	60 min	60 min	0.14	-0.14	0.41

SB, sedentary behavior; MVPA, moderate-to-vigorous physical activity; BMI, body mass index; WC, waist circumference; BF%, body fat percentage; HDL-C, high-density lipoprotein cholesterol; Non-HDL-C, non-high-density lipoprotein cholesterol; LDL-C, low-density lipoprotein cholesterol; TG, triglyceride; HOMA2-IR, homeostatic model assessment of insulin resistance; HOMA2-S, homeostatic model assessment of insulin sensitivity; HOMA2-β, homeostatic model assessment of beta cell function; SBP, systolic-blood pressure; DBP, diastolic-blood pressure; CRS, cardiometabolic risk score; CI, confidence interval. Boldface represents the statistical significance ($p < 0.05$).

[a]Adjusted for daily awake time (hours), accelerometer valid days, age, smoking status, and body mass index (BMI) (except when BMI was the dependent variable).

[b]Data transformed from natural log scale for better interpretation.
